# 

**Published:** 1895-01-05

**Authors:** 


**Notices of Births, Deaths, and Marriages** are inserted in this column at a charge of 2s. 6d. for an announcement not exceeding four lines.

**Notice to Advertisers and Subscribers.**

All Advertisements, Orders for Copies of THE HOSPITAL, and Business Communications should be addressed to **The Manager, NOT TO THE EDITOR,** THE HOSPITAL, 428, Strand, London, W.C.

The Cost of Subscription, post free, is as follows:—Per week, 2½d.; per quarter, 2s. 8d.; per half-year, 5s. 3d.; per annum, 10s. 6d. Foreign:—Per Annum, 15s. 2d.; payable, in all cases, in advance.

### NOTICE TO CORRESPONDENTS.

All MS., Letters, Books for Review, and other matters intended for the Editor should be addressed THE EDITOR, THE LODGE, PORCHESTER SQUARE, LONDON, W.

The Editor cannot undertake to return rejected MS., even when accompanied by stamped directed envelope.

**"Burdett's Hospital Annual," 1895,**

IN ACTIVE PREPARATION.

Sixth year of publication. About 900 pp. Scarlet cloth, gilt lettered. Price 5s. A most complete guide to British, Colonial, Indian, and American Hospitals, Dispensaries, Nursing and Convalescent Institutions, and Asylums. A few copies of the "Annual" for 1894 may still be obtained on application to the Publishers, THE SCIENTIFIC PRESS (Limited), 428, Strand, London, W.C.

**NOTICE.—The Index for Vol. XVI. (ending September 24th, 1894) is on sale at the Office of this Journal, Price 2½d., post free.**

### Scraps and Gleanings.

A gift of pheasants has been made to the patients at the Hospital for Consumption at Brompton by the Prince of Wales.

The annual report of the Victoria Infirmary, Glasgow, shows an increase of 214 in the number of patients treated in 1894 as compared with the previous year.

Great improvements have recently been carried out at the Carnarvonshire and Anglesey Infirmary, Bangor, and the reopening ceremony took place early in December.

The amounts paid into the Hull Hospital Saturday Fund since October 28th now bring the total up to £708 8s. 3d. There are still several collections not yet paid in to the treasurers.

The annual report of the Dublin Orthopædic Hospital emphasises the fact that the income for the twelve months does not cover the expenditure. Funds are most earnestly needed to cope with the growing needs of this most deserving charity.

It appears that in the early stages of the proposal to erect a new infirmary at Selby Oak, it was estimated that something like £10,000 would suffice for the scheme. Now we learn that the first contract received for this institution is nearly £18,000.

The Empress Frederick has given a present of a bust of the late Prince Waldemar of Prussia to the children's ward of the Throat Hospital, Golden Square. The ward was named after the Empress on the occasion of her recent visit to the hospital.

Special services were held last month in the Macclesfield churches, and in the various places of worship in the town and district, in aid of the funds of the local infirmary, the collections accruing from the various sources having materially assisted the institution.

The Nottingham Hospital Saturday Committee have arranged to invite all the public works of the town and neighbourhood to raise subscriptions for the General Hospital and other local medical charities. The subscriptions thus obtained are to the committee on February 16th.

The sum of £25 has been received by the secretary of the South Charitable Infirmary, Cork, the bequest of the late Mr. J. H. Bennett. It is further stated that in the event of certain contingencies, one-third of a sum of £4,000, a portion of the money left by the deceased, will go to the benefit of the institution.

The National Hospital for Consumption at Dublin held its second annual meeting early this month. The hospital is still in course of erection, but three blocks are already roofed in. Several generous donations have been promised, and subscriptions are being appealed for towards the annual support of the institution.

The Managing Committee of the Cork Street Fever Hospital at Dublin held their usual meeting on December 6th. In the report it was stated that among the numerous admissions to the hospital of patients suffering from small-pox, which still continues prevalent, not one vaccinated person has been admitted with the disease.

### BOOKS RECEIVED.

H. K. LEWIS.

"The Aseptic Treatment of Wounds." By Dr. C. Schimmelbusch. Translated by Alfred Theodore Rake, M.B.

"The Anatomy of the Nasal Cavity." By Dr. A. Onodi. Translated by Dr. Clair Thomson, M.D.

"The Prevention of Small-pox." By Edgar M. Crookshank, M.B.

"The Public, the Profession and Medical Charities." By Robert Boxall, M.D.

SCIENTIFIC PRESS.

"Helps in Sickness and to Health." By Henry C. Burdett.

JOHN WRIGHT, BRISTOL.

"Epitomes of Modern Surgical Progress." By E. Henry Fenwick, F.R.C.S.

J. AND A. CHURCHILL.

"The Prevention of Epidemics, &c." By Roger McNeill, M.D.Edin.

**Periodicals and Pamphlets.**—Medical Record, Provincial Medical Journal, Clinical Journal, Guy's Hospital Gazette, Leeds Hospital Magazine, Child's Guardian, The Sun, Revue Générale des Sciences, Homœopathic Review, Medical Press and Circular, Public Health, Toilers of the Deep, Vegetarian, Merrie England, The Epicure, Charlotte Medical Journal, Forward, Newsagent and Bookseller, The Publishers' Weekly, The Literary News, Sunshine, Charity Organisation Review, The Health Record, Industries and Iron, Cornhill Magazine, Humanitarian, Christian World, Our Christmas Hamper, The Planter, London, Reich's Medicinal Anzeiger, The Zenana, Service for the King.

### THE STUDENT OF MEDICINE.

### VACANCIES.

The following vacancies are announced this week, the amount of salary in each case being given after the name of the institution:

### Resident Medical Officers.

Birmingham and Midland Free Hospital for Sick Children.—Resident Medical Officer and Resident Surgical Officer. Salary, £70 and £50 per annum, respectively, with board, washing, and attendance in the institution. Applications to the Secretary, Children's Hospital, Steelhouse Lane, Birmingham, by January 8th.

Devon and Exeter Hospital, Exeter.—Assistant House Surgeon; doubly qualified, and unmarried. Salary, £40 per annum, with board and lodging. Applications to Albert E. Boyce, Secretary, by January 7th.

Parochial Board of Barony Parish of Glasgow.—Senior Assistant to the Medical Superintendent of the Barony Parochial Asylum, Woodilee, Lengie, near Glasgow. Salary £200 per annum, with board, apartments, &c. Applications, marked "Woodilee Medical Assistant," to Jas. R. Morton, Acting Inspector of Poor, Barony Parish Chambers, 38, Cochrane Street, Glasgow, by January 12th.

West Herts Infirmary, Hemel Hempstead.—House Surgeon and Dispenser, doubly qualified and unmarried. Appointment for two years. Salary, £100 per annum, with board, furnished rooms, fire, lights, attendance, and washing. Applications to the Secretary by January 17th.

### Non-Resident Medical Officers.

Eltham College, Kent (Royal Naval School).—Medical Officer. Applications to the Rev. the Bursar by January 5th.

Hospital for Sick Children, Great Ormond Street, W.C.—Two Assistant Physicians; must be F. or M.R.C.P.Lond. Applications to the Secretary by January 14th.

London County Council.—Pathologist to the London County Asylum. Salary, £700 per annum, with travelling expenses. Applications, on forms to be obtained at the office of the Committee, endorsed "Application for Pathologist," to R. W. Partridge, Clerk to the Asylums Committee, London Asylums Committee Office, 21, Whitehall Place, S.W., by Ten o'clock on January 5th.

London Hospital Medical College, Turner Street Mile End.—Lectureship on Organic Chemistry. Salary not less than £175 a-year. Applications to Munro Scott, Warden, by January 10th.

Norfolk and Norwich Hospital.—Assistant to the House Surgeon, doubly qualified. Appointment for six months. Board, lodging, and washing provided. Applications to the House Surgeon by January 5th.

Westminster Hospital. — Fourth Assistant Surgeon. Must be F.R.C.S.Eng. Candidates must transmit certificate of age, and attend the House Committee on January 1st.

### THE STUDENT OF MEDICINE—*continued.*

### UNIVERSITIES AND COLLEGES.

#### UNIVERSITY OF LONDON.

M.B. EXAMINATION FOR HONOURS.

MEDICINE.—First Class: A. H. Leete, B.Sc. (Scholarship and Gold Medal), Guy's Hospital; A. E. Russell (Gold Medal), St. Thomas's Hospital; G. E. Manning, Guy's Hospital; F. J. Poynton, St. Mary's Hospital; J. S. Collier, B.Sc., St. Mary's Hospital; G. B. Hunt, University College; J. Stephenson, B.Sc., Owens College and Manchester Royal Infirmary; F. J. Steward, Guy's Hospital; J. H. Fisher, St. Thomas Hospital; R. A. Young, B.Sc., Middlesex Hospital. Second Class: E. C. Taylor, Guy's Hospital; J. S. Bolton, B.Sc., University College; E. M. Hainworth, B.Sc., St. Thomas's Hospital; F. G. Hopkins, B.Sc., Guy's Hospital; A. Stanley, St. Mary's Hospital; G. A. Cowen, London Hospital; H. Davies, Guy's Hospital; Alice Mary Corthorn, London School of Medicine for Women; W. E. Lee, St. Bartholomew's Hospital; M. H. Raper, Middlesex Hospital; J. S. Sloane, B.Sc., St. Bartholomew's Hospital; E. Smith, St. Thomas's Hospital. Third Class: C. S. Wallace, St. Thomas's Hospital; Lizzie Bennett London School of Medicine for Women; Susan Anne Hughes, London School of Medicine for Women and Royal Free Hospital; R. H. Steen, Queen's College, Belfast, and St. Mary's Hospital; A. C. Hovenden, Guy's Hospital; E. W. Spink, Yorkshire College; J. W. Haines, St. Bartholomew's Hospital; W. Shears, St. Bartholomew's Hospital; Maud Mary Chadburn, London School of Medicine for Women and Royal Free Hospital.

OBSTETRIC MEDICINE.—First Class: W. E. Lee (Scholarship and Gold Medal), St. Bartholomew's Hospital; *C. S. Wallace (Gold Medal), St. Thomas's Hospital; F. J. Steward, Guy's Hospital; E. C. Taylor, Guy's Hospital. Second Class: F. J. Poynton, St. Mary's Hospital; A. E. Russell, St. Thomas's Hospital; J. H. Fisher, St. Thomas's Hospital; A. N. Weir, B.Sc., St. Bartholomew's Hospital. Third Class: G. H. Cowen, London Hospital; Elizabeth Jane Moffett, B.Sc., London School of Medicine for Women and Royal Free Hospital; E. H. Houfton, Yorkshire College. * Obtained the number of marks qualifying for the University Scholarship.

FORENSIC MEDICINE.—First Class: J. S. Collier (Scholarship and Gold Medal), St. Mary's Hospital; J. S. Bolton (Gold Medal), University College; M. H. Raper, Middlesex Hospital; G. E. Manning, Guy's Hospital; F. J. Steward, Guy's Hospital; A. Stanley, St. Mary's Hospital: Y. H. Mills, London Hospital. Second Class: E. E. Murray, University College; T. Warner, King's College; H. P. Meakin, St. Bartholomew's Hospital. Third Class: F. D. Harris, St, Mary's Hospital; F. G. Hopkins, Guy's Hospital; Elizabeth Jane Moffett, London School of Medicine for Women and Royal Free Hospital.

#### UNIVERSITY OF EDINBURGH.

The following gentlemen have been appointed office-bearers of the Edinburgh University Union for the ensuing session: President—S. J. Aarons. Honorary Secretary—James G. C. Scott. Treasurer: W. A. Wood, C.A. Auditor—H. Hay Brown, C.A. Committee of Management—Professor T. Annandale, Professor Butcher, Dr. Musgrove, Dr. J. G. Cattanach, Mr. C. W. Cathcart, F.R.C.S., Mr. James A. Clyde, LL.B., Messrs. W. J. Garbutt, A. McArthy Morrogh, J. W. Tullo, B.Sc., D. Waterston, R. D. Melville, and R. D. Story.

#### FACULTY OF PHYSICIANS AND SURGEONS OF GLASGOW.

At the December meeting of the Faculty held on Monday, December 3rd, George Balfour Marshall, M.D., Woodside Crescent, Glasgow, and Charles J. Renshaw, M.D., Ashton-upon-Mersey, having passed the necessary examination, were admitted Fellows of the Faculty.

### "THE HOSPITAL."

London: THE SCIENTIFIC PRESS (Limited), 428, Strand, W.C.

Printed by W. H. and L. COLLINGRIDGE, City Press, 148 and 149, Aldersgate Street, London, E.C.; and Published by the Proprietors, THE SCIENTIFIC PRESS (LIMITED), at 428, Strand, London, W.C.

To be had of all Booksellers and Newsagents in the United Kingdom, and at all the Railway Bookstalls of Messrs. W. H. Smith and Son.

### THE SCIENTIFIC PRESS LIST.

The SCIENTIFIC PRESS beg to announce that they have been appointed agents in Great Britain for the sale of the publications of the W. T. Keener Co., of Chicago, and have now the following works in stock:—

INTESTINAL SURGERY. N. SENN, M.D. 8vo. cloth, illustrations. Price 10s. net.

EXPERIMENTAL SURGERY. N. SENN, M.D. 8vo., cloth, illustrations. Price 20s. net.

STRICTURE OF THE URETHRA. G. F. LYDSTON, M.D. 8vo., cloth, plates and illustrations. Price 12s. net.

VARICOCELE AND ITS TREATMENT. G. F. LYDSTON, M.D. 8vo., cloth, illustrations. Price 5s. net.

ETIOLOGY OF OSSEOUS DEFORMITIES OF THE HEAD, FACE, JAWS, AND TEETH. E. S. TALBOT, M.D. Third Edition. 8vo., cloth, 461 illustrations.

RECTAL AND ANAL SURGERY. E. ANDREWS, M.D. Third Edition, 8vo. 6s. net.

TECHNIQUE OF POST-MORTEM EXAMINATION. L. HEKTOEN, M.D. 41 illustrations. 8s. net.

CHEMICAL ANALYSIS FOR BEGINNERS. RÜDORFF, GIBSON, and MEAZEL. 8vo., cloth.

---

"Mr. Burdett's monumental work."—*The Times.*

In Four Volumes, Crown 4to., cloth, gilt bevelled edges, Profusely Illustrated with Plans and Designs; together with a portfolio containing some Hundreds of Plans. Price £8 8s. complete.

### Hospitals and Asylums of the

WORLD. Their Origin, History, Construction, Administration, Management, and Legislation; with Plans of the Chief Hospitals, Asylums, Convalescent Institutions, Special and Infectious Hospitals, Nurses' Homes, and Medical School Buildings, in addition to those of all the Hospitals of London in the Jubilee Year of Queen Victoria's Reign, accurately drawn to a uniform scale. By HENRY C. BURDETT. Vols. I. and II.—ASYLUMS: History and Administration, Construction, Plans, and Bibliography. Price £4 10s. Vols. III. and IV.—HOSPITALS: History and Administration, Construction, Plans, and Bibliography. With Portfolio containing some hundreds of Plans. Price £6. The entire work complete £8 8s.

Of the small edition printed a limited number of copies still remain, and Institutions, Libraries, and Clubs have therefore an opportunity to add this remarkable work to their libraries which cannot much longer continue. A limited number of Portfolios of Plans will be supplied separately at £3 3s. each.

"An era in the history of hospital literature."—*Spectator.*
"Contains as much matter as an ordinary library."—*Guardian.*
"Indispensable to any Committee."—*Times.*

Sixth Year of Publication, about 900 pp., Crown 8vo., Cloth gilt.

Price 5s. [*In Preparation.*

### Burdett's Hospital and Chari-

TIES ANNUAL, BEING THE YEAR-BOOK OF PHILANTHROPY, 1895. Containing a Review of the position and Requirements of the Voluntary Charities, and an Exhaustive Record of Hospital Work for the Year. It will also be found to be the most useful and reliable Guide to British, Colonial, and American Hospitals, Dispensaries, Nursing and Convalescent Institutions and Asylums. Edited by HENRY C. BURDETT.

"An excellent book of reference."—*British Medical Journal.*

"No one who takes up this book can fail to be impressed with its value."—*The Lancet.*

Demy 8vo., cloth gilt, 6s.

### The Uniform System of Ac-

COUNTS, AUDIT, AND TENDERS for HOSPITALS and INSTITUTIONS, with CERTAIN CHECKS upon EXPENDITURE. Also the Index of Classification. Compiled by a Committee of Hospital Secretaries, and adopted by a General Meeting of the same, Jan. 18th, 1892. And certain Tender and other Forms for securing economy. By HENRY C. BURDETT. All having to do with hospital accounts will find much valable information in this work. Secretaries making up returns for participation in the donations of the Metropolitan Hospital Sunday Fund will find it invaluable. It will be of special interest also to the officials of all institutions—treasurers, secretaries, and auditors—as it expounds practically the uniform system which is now being widely adopted as a means of saving labour and bringing all systems of accounts into uniformity.

### Account Books for Institu-

TIONS: Designed in accordance with the Uniform System of Accounts for Hospitals and Institutions. By HENRY C. BURDETT. Being a complete set of Royal, Foolscap, and Double Foolscap Account Books, ruled in accordance with the uniform system adopted by the Metropolitan Hospital Sunday Fund. Designed and constructed for the convenience and assistance of secretaries who present annual returns for participation in the Hospital Sunday Fund grants. These books are ruled so that the various descriptions of receipts and expenditure, &c., &c., may be entered uniformly under the special headings and in the columns prepared for them. By their use the labour of hospital secretaries is reduced to a minimum and the accounts of all public institutions can be kept in a uniform manner. Every detail has been minutely dealt with, and all possible entries provided for. Printed headings and divisions render the system perfectly plain and lessen the work of keeping the accounts in a very large degree.

(Descriptive Price List post free on application.)

Royal 8vo., 500 pp., 60 Illustrations, cloth gilt. Price one guinea net.

### Hospitals, Dispensaries, and

NURSING: Being Transactions of Section III. International Congress of Charities, Correction, and Philanthropy, held in Chicago, 1893.

This valuable collection of papers is published in one handsome volume and comprises a variety of subjects, each of which is dealt with by a representative and leading authority.

Now ready, demy 8vo., with 2 Plates, handsome cloth gilt, over 400 pp., price 12s. 6d. net.

### Medical History from the

EARLIEST TIMES. By E. T. WITHINGTON, M.A., M.B.Oxon. This important history of Medicine from the earliest times has been treated in an exhaustive and thorough manner. It comprises sixty-five chapters and seven appendices, commencing with Medicine in Pre-historic Times, and ending with the Origin of Modern Medicine.

"One of the best attempts that has yet been made in the English language to present the reader with a concise epitome of the history of medicine, and as such we cordially commend it."—*Glasgow Medical Journal.*

"This learned but fascinating volume may be recommended to all readers."—*Daily Chronicle.*

### The Johns Hopkins Hospital Reports.

The Scientific Press (Limited) have been appointed sole agents for the sale of the above Reports in the United Kingdom. The under-mentioned volumes (with the exception of Vol. I.) are stocked at 428, Strand, London, W.C., and can be supplied at once.

THE JOHNS HOPKINS HOSPITAL REPORTS.

Vol. I. is in the press. It will contain the studies from the Pathological Laboratory.

Vol. II. 4to, cloth, 570 pp., 28 Plates and many Illustrations. Price 21s. net. Contents: Medical Report for 1890, I.—Medical Report for 1890, II.—Report in Gynæcology, I.—Report in Surgery, I.—Report in Neurology, I.—Report in Pathology, I.

Vol. III., 4to, cloth, 766 pp., 69 Plates and many Illustrations, price 21s. net. Contents: Report in Pathology, II.—Report in Pathology, III.—Report in Gynæcology, II. (The Report in Gynæcology, II., of Vol. III., can also be obtained separately, price 12s. 6d. net.)

Vol. IV. now in progress. Subscription for the volume, 21s. net. The Report on Typhoid Fever now ready, 4to, 160 pp., with Charts, &c., price 5s. net. The Report on Neurology is just ready, 4to, 130 pp., with Plates, &c., price 7s. 6d. net. List, with complete contents, sent on application.

IMPORTANT NEW HANDBOOK OF MIDWIFERY.

18mo., profusely illustrated with original cuts, tables, &c.; about 260pp., handsomely bound in leather, price 6s.

### A Practical Handbook of Mid-

WIFERY. By FRANCIS W. N. HAULTAIN, M.D. A practical manual produced in a portable and convenient form for reference. Both to the Profession and to Students it is recommended for its compactness, conciseness, and clearness.

"The book is one of the best of its kind, and is well fitted to perform the functions its author claims for it."—*Edinburgh Medical Journal.*

"We can heartily recommend this book as a practical help."—*Glasgow Medical Journal.*

"No practical handbook published recently will . . . be more welcome to students than this on Midwifery just issued."—*Edinburgh Student.*

Now ready, demy 8vo., about 150 pp., profusely Illustrated, with Coloured and other Plates and Drawings, price 6s. net.

### Lectures on Genito-Urinary

DISEASES. By J. C. OGILVIE WILL, M.D., C.M., F.R.S.E., Consulting Surgeon to the Aberdeen Royal Infirmary, and Examiner in Surgery in the University of Aberdeen. Contents: Urethral Fever and Catheter Fever—Treatment of Retention of Urine—Gleet and its Treatment—On Varicocele—On Hydrocele—The Treatment of Syphilis—Appendix—Prescriptions for Syphilis, &c., &c.

"We have no hesitation in recommending Dr. Ogilvie Will's work."—*Practitioner.*

"No one can peruse them without deriving much valuable information."—*Manchester Medical Chronicle.*

London: THE SCIENTIFIC PRESS (Limited), 428, Strand, W.C.

